# Fingerprint of the oxido-reductase ERO1: A protein disulfide bond producer and supporter of cancer

**DOI:** 10.1016/j.bbcan.2023.189027

**Published:** 2023-11-24

**Authors:** Ester Zito, Luca Guarrera, Yvonne M.W. Janssen-Heininger

**Affiliations:** a Istituto di Ricerche Farmacologiche Mario Negri IRCCS, Milan, Italy; b Department of Biomolecular Sciences, University of Urbino Carlo Bo, Urbino, Italy; c Departments of Pathology and Laboratory Medicine, University of Vermont College of Medicine, Burlington, VT, USA

**Keywords:** ERO1 alpha, Endoplasmic reticulum stress, UPR (unfolded protein response), Metastasis, Hypoxia, Angiogenesis, cancer

## Abstract

Endoplasmic reticulum oxidoreductin 1 (ERO1) alpha (ERO1A) is an endoplasmic reticulum (ER)-localized protein disulfide oxidoreductase, involved in the disulfide bond formation of proteins. ERO1’s activity in oxidative protein folding is redundant in higher eukaryotes and its loss is well compensated. Although it is dispensable in non-cancer cells, high ERO1 levels are seen with different cancers and predict their malignant phenotype. ERO1 fosters tumor aggressiveness and the response to drug therapy in hypoxic and highly metastatic tumors. It regulates vascular endothelial growth factor (VEGF) levels, oxidative folding and *N*-glycosylation in hypoxic conditions, boosting tumor fitness and angiogenesis on multiple levels. In addition, ERO1 regulates protein death ligand-1 (PD-L1) on tumors, interfering with the related immune surveillance mechanism, hence acting on the tumors’ response to immune check-point inhibitors (ICI). This all points to inhibition of ERO1 as an effective pharmacological tool, selectively targeting tumors while sparing non-cancer cells from cytotoxicity. The critical discussion here closely examines the molecular basis for ERO1’s involvement in tumors and ERO1 inhibition strategies for their treatment.

## 1. Introduction

Protein folding of secretory and membrane proteins, which usually includes disulfide bond formation and asparagine (*N*)-linked glycosylation, occurs in the endoplasmic reticulum (ER) lumen in a co-translational or post-translational manner [4,9]. This fine-tuned process is regulated by the activity of enzymes which respond to ER stress in a coordinated fashion, triggering a homeostatic response, referred to as the unfolded protein response (UPR), aimed at re-establishing ER homeostasis [61]. Among these enzymes ERO1, through its partner in the redox reaction, protein disulfide isomerase (PDI) introduces disulfide bonds in proteins, leading to oxidative protein folding [71,72]. ERO1 is a flavin adenine dinucleotide (FAD)-containing protein which, through a relay of electrons, restores the oxidized state of PDI, making it available for a new cycle of disulfide bond formation in proteins. ERO1 couples this redox reaction with the two-electron reduction of O_2_, producing stoichiometric amounts of H_2_O_2_ [19,54]. Thus, the final balance of the reaction is the production of one molecule of H_2_O_2_ for every disulfide bond that is formed. Importantly, it has been estimated that ERO1 activity accounts for 25% of the H_2_O_2_ produced during protein translation [55]. By virtue of its role as a potent H_2_O_2_ producer, ERO1 was also considered a mediator of maladaptive UPR, inducing cell death [74].

Unlike yeast (in which ERO1 deficiency is lethal) in higher eukaryotes ERO1’s protein disulfide oxidase activity is compensated by other enzymes, such as PRDX4, GPX8 and GPX7, that participate in protein disulfide bond formation while metabolizing H_2_O_2_. Compensation for loss of ERO1 explains why mice lacking both ERO1 isoforms (alpha and beta) show only a delay in disulfide bond formation and subtle functional defects [36,71–74].

Cancer cells encounter constant ER stress due to the high proliferation rate and conditions such as hypoxia, shortage of nutrients, and genetic mutations (*e.g.*; those in KRAS [12]). As a consequence, the UPR is triggered, activating corrective measures to help the cancer cells survive and thrive. The adaptive UPR acts in tumors through a cell-autonomous mechanism contributing to the thriving and survival of the cells, and through cell non-autonomous mechanisms driving angiogenesis, for example, by increasing the master regulator of angiogenesis, vascular endothelial growth factor (VEGF), and the dysregulation of immune cells in the tumor microenvironment (TME) [7,8,32,42,62].

In cancer, ERO1 alpha (henceforth ERO1) is part of the adaptive UPR, which helps cancer cells to cope with the oxidative protein folding of the high load of new nascent proteins [74]. High ERO1 levels are reported in different cancers and are predictive of their malignant phenotype and worse clinical outcomes [69,22,49,50,65,66].

Functionally, ERO1 activity interferes with mitochondrial bioenergetics, the redox status and the *N*-glycosylation of mediators of angiogenesis, and is thus involved in the tumor metabolic rewiring and angiogenesis, favoring metastasis and cancer aggressiveness in general [39,68]. ERO1 is also involved in regulating PD-L1 [51,60] on tumors, enhancing the tumor-related immunosuppressive mechanisms and the response to ICI.

Here, we review the cancer-related mechanisms and the molecular signals through which ERO1 influences the steps of oncogenesis, with a focus on the therapeutic potential of targeting ERO1 in cancer.

## 2. ERO1 activity in cells

The role of ERO1 as an enzyme that takes part in protein disulfide oxidation is conserved through eukaryotes. In simple eukaryotes, such as yeast, ERO1 is present as a single isoform and its deficiency is lethal [11,40]. Mammals, however, have two ERO1 isoforms encoded by separate genes. ERO1 alpha is widely expressed, while ERO1 beta expression is restricted to the stomach and the endocrine pancreas [71]. Surprisingly, mammalian cells deficient in both ERO1 isoforms present only a kinetic delay in disulfide bond formation, and compound ERO1 alpha and beta mutant mice are viable and fertile [71], suggesting there are ERO1-independent pathway(s) for disulfide bond formation [72,73]. Since ERO1 activity generates stoichiometric amounts of H_2_O_2_ it is considered a source of oxidative stress and a mediator of the maladaptive UPR [71–74]. In support of this, experiments in *Caenorhabditis elegans* report that the knockdown of *ero-1* lowers the levels of endogenous peroxides in ER-stressed worm tissue, while prolonging their lifespan [16,18] A similar scenario is apparent in mammals, where *Perk*^−/−^ cells accumulate peroxides during ER stress, while the interference of the ER oxidase ERO1 abrogates this accumulation [16].

In aggregate, these observations point to the compensation of ERO1 activity in mammals, and suggest not only that cells might safely survive without ERO1 but also that, in certain conditions, its inhibition might even be advantageous.

## 3. ERO1 expression in cancer

**Fig. 1.**
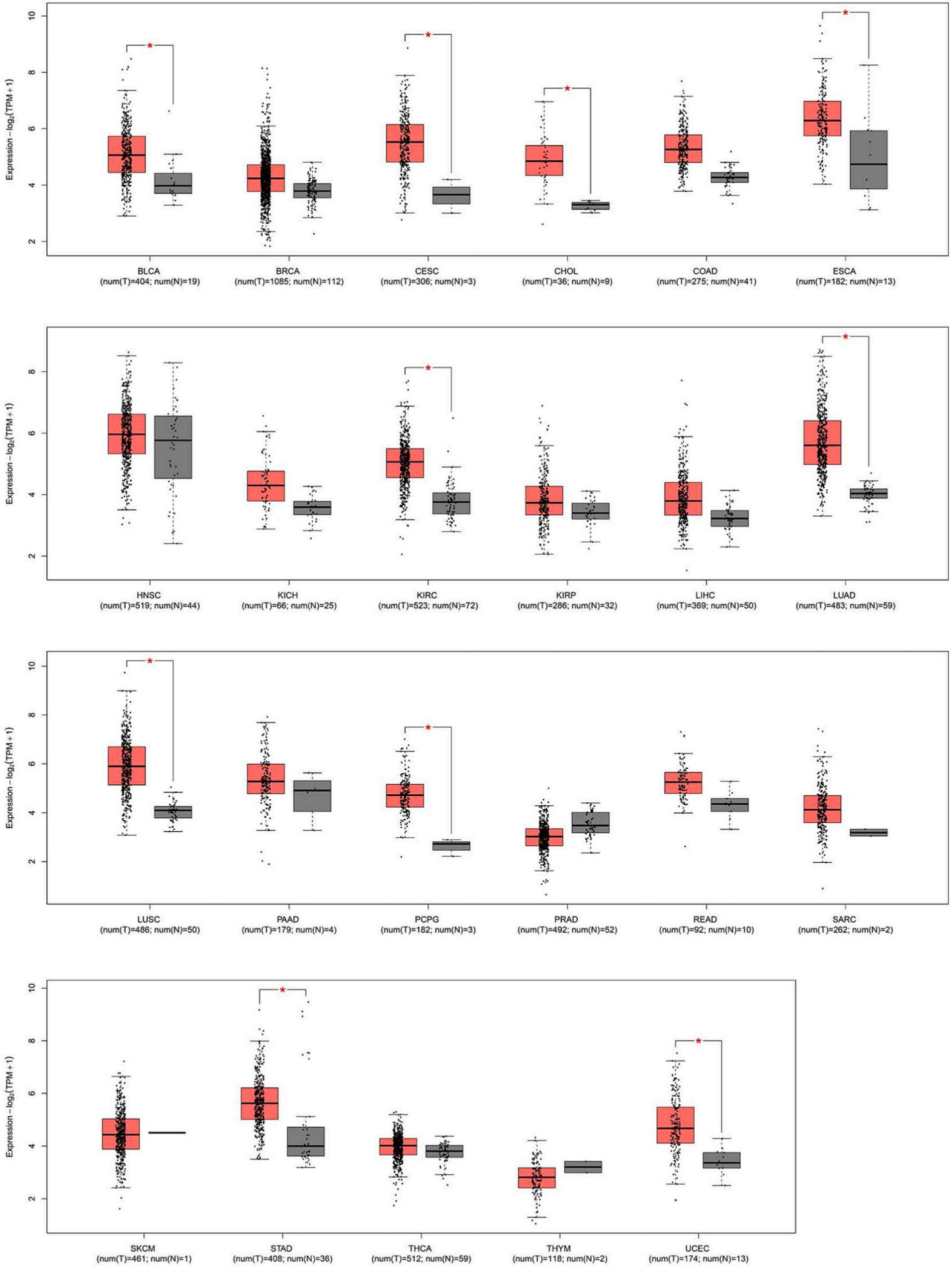
ERO1 expression in tumors and non-tumor tissues. Box plot of the different expression of the ERO1 gene across multiple tumor types, where tumor samples are depicted in red and healthy tissues adjacent to the tumor in gray. On the x-axis, each box corresponds to a specific cancer type, while the y-axis represents the gene expression values normalized as log2(TPM + 1). TPM, or Transcripts Per Million, is a method of normalization used to account for RNA composition, providing a measure of gene expression levels in a given sample. Data were sourced from the GEPIA2 platform [52], utilizing the TCGA dataset [53]. Red: tumor, Gray: adjacent normal tissue; BLCA: Bladder Urothelial Carcinoma; BRCA: Breast Invasive Carcinoma; CESC: Cervical squamous cell carcinoma and endocervical adenocarcinoma; CHOL: Cholangiocarcinoma; COAD: Colon adenocarcinoma; ESCA: Esophageal carcinoma; HNSC: Head and Neck squamous cell carcinoma; KICH: Kidney Chromophobe; KIRC: Kidney renal clear cell carcinoma; KIRP: Kidney renal papillary cell carcinoma; LIHC: Liver hepatocellular carcinoma; LUAD: Lung adenocarcinoma; LUSC: Lung squamous cell carcinoma; PAAD: Pancreatic adenocarcinoma; PCPG: Pheochromocytoma and Paraganglioma; PRAD: Prostate adenocarcinoma; READ: Rectum adenocarcinoma; SARC: Sarcoma; SKCM: Skin Cutaneous Melanoma; STAD: Stomach adenocarcinoma; THCA: Thyroid carcinoma; THYM: Thymoma; UCEC: Uterine Corpus Endometrial Carcinoma

*ERO1* RNA levels (TCGA dataset) from tumors and corresponding normal tissues indicated that the majority of tumors had high *ERO1* expression, suggesting a potential role of ERO1 in tumor fitness (Fig. 1).

**Fig. 2.**
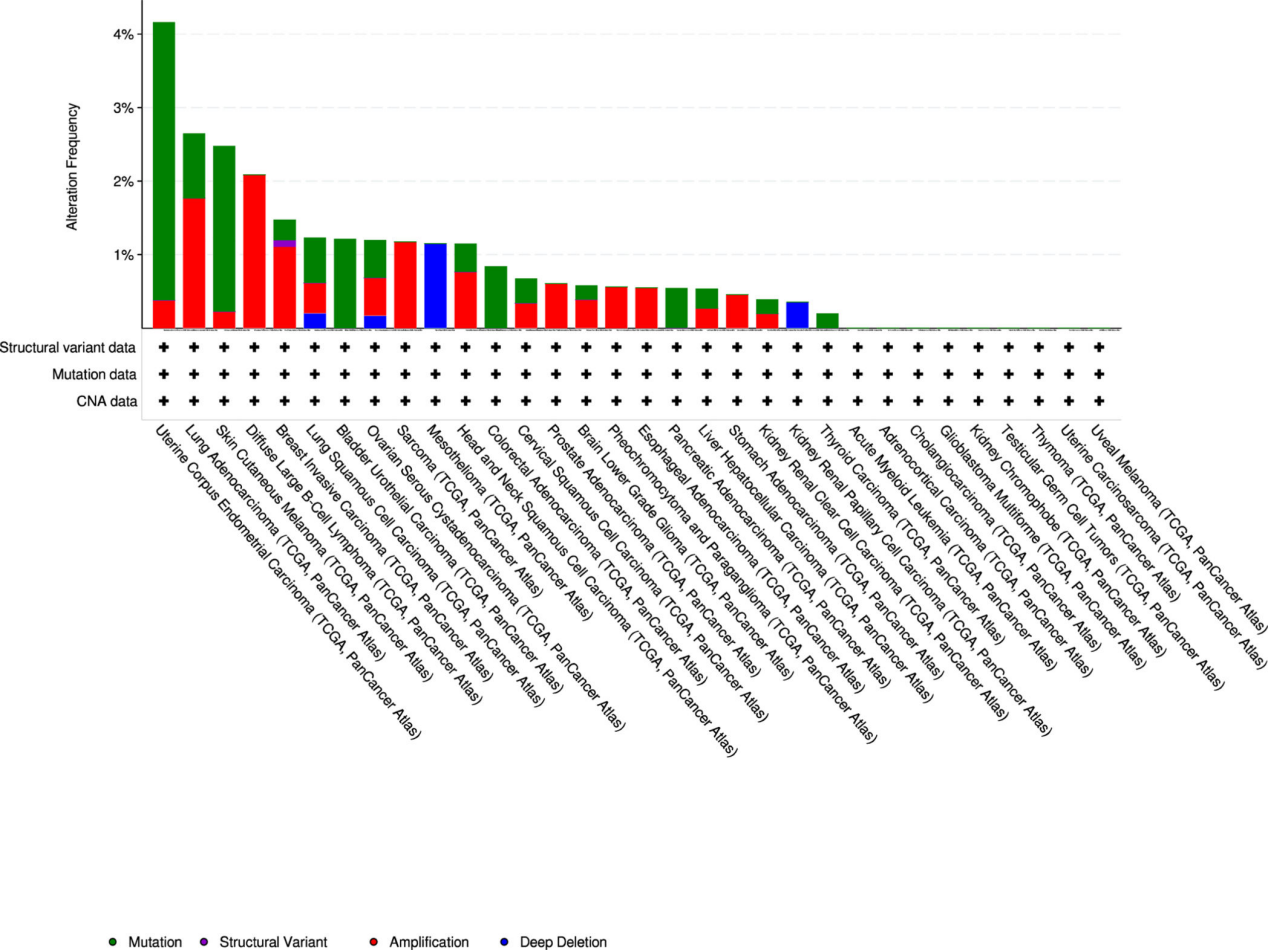
Genetic alterations in *ERO1* gene in different cancer types. ERO1 genetic mutations were analyzed using the cBio Cancer Genomics Portal (http://cbioportal.org). The following genetic changes were considered: single nucleotide mutation (green), gene amplification (red), structural variant (purple) and deep deletion (blue). Alteration frequency (%) is shown on the Y axis. (For interpretation of the references to colour in this figure legend, the reader is referred to the web version of this article.)

We also analyzed the genetic mutations in *ERO1* gene in different cancer types (TCGA PanCancer ATLAS) and analyzing 10,953 patients using the cBio Cancer Genomics Portal [6]. The genetic alterations in *ERO1* gene were mainly missense, gene amplification and fusion (in the breast) type mutations, seen most in the uterus, lung, skin, B cell, and breast cancer (Fig. 2 and Table 1). Although it remains unclear whether and how the missense mutations affect ERO1 activity, the frequent gene amplification mutations of *ERO1* further support a role for its overexpression in cancer.

**Fig. 3.**
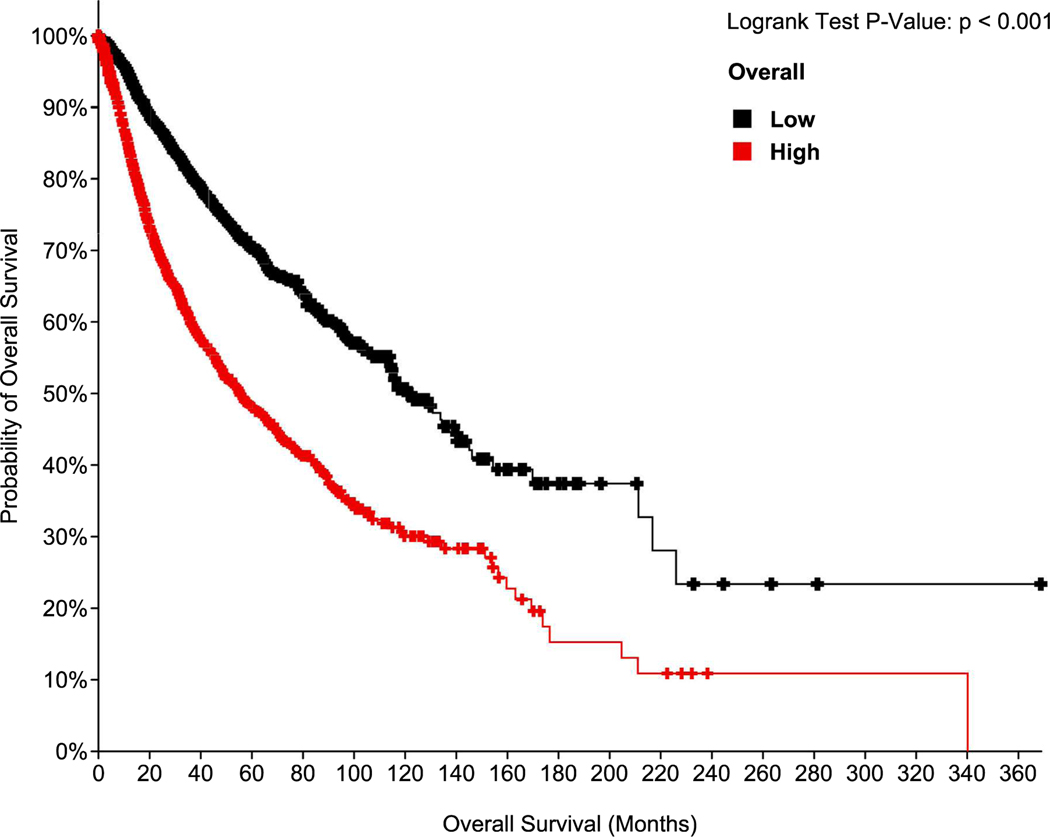
Correlation between ERO1 expression levels in cancer and overall survival. Kaplan-Meier plot depicting overall survival of cancer patients treated and stratified in quartiles for gene expression levels of ERO1. Statistical significance was assessed using a log-rank test. Data were from the Pan TCGA data set (TCGA PanCancer ATLAS) with 10,953 patients.

From the perspective of ERO1’s impact on the life expectancy of cancer patients, analysis of data that were sourced from the GEPIA2 platform [52], utilizing the TCGA dataset [53] indicates that higher expression of ERO1 (in quardle Q1 *vs* Q4), in tumors correlates with lower overall survival (Fig. 3).

Interrogation of Cancer Cell Line Encyclopedia datasets indicates that in breast cancer, *ERO1* mRNA levels were higher in basal cancer cells, which are mostly aggressive triple-negative breast cancer (TNBC). *ERO1* mRNA from breast cancer tissues from the Cancer Genome Atlas (TCGA) database were significantly upregulated in basal cancer compared to luminal and normal tissue, confirming the cell results in tissue [56]. In addition, our bioinformatics analysis from the Metastatic Breast Cancer project indicates an inverse correlation between ERO1 (high) levels in primary aggressive TNBC and the (shorter) time at which distant metastases are detected, suggesting a pivotal role of ERO1 in conferring an aggressive phenotype [56]. These findings support the observations that ERO1 overexpression is associated with different cancers, and poor prognosis for some of them (*e.g.*, breast, multiple myeloma, pancreatic, cervical, liver, prostate and gastric cancers) [13,14,17,22,50,65,67].

ERO1 expression is upregulated by ER stress and hypoxia – *i.e.*, a scarcity of oxygen [34], two hallmarks of malignant cancers [31,64]. Oxygen levels can fall to 0.01% in tumor cells, implying that hypoxia is a hallmark common to many solid tumors associated with poor clinical outcomes. Under low-oxygen tensions, hypoxia-inducible factor-1 (HIF-1) is activated and promotes the transcription of angiogenic factors (among these VEGF) by recognizing a consensus hypoxia response element in their promoter, triggering tumor angiogenesis and aggressiveness [44]. Experimentally, the analysis of ERO1 levels in basal breast cancer cells cultured under hypoxic conditions (*i.e.*, O_2_ lower than 0.1%) confirmed up-regulation of ERO1 mRNA and protein levels [56]. These findings suggest ERO1 might serve as a potential biomarker of aggressive cancers. Here, we discuss the molecular signaling modulated by ERO1 in oncogenesis.

## 4. The role of ERO1 in tumor metabolic rewiring

Mitochondria support multiple processes of tumors such as their resistance to adverse environmental conditions, including chemotherapy, promoting their spread. Although it has long been thought that the bioenergetics of cancer cells rely mostly on glucose, in the last few years it has become clear that oxidative phosphorylation (OXPHOS), a mitochondria-based process of ATP production, helps cancers thrive by triggering tumor resistance to chemotherapy, and aggressiveness [24, 41, 43]. Molecular signaling, improving mitochondrial bioenergetics, might therefore, influence oncogenesis, supporting it. ERO1 is enriched in a region of the ER in contact with mitochondria, referred to as mitochondria-associated membranes (MAMs) [1], and in this strategic location, it improves ER-mitochondrial Ca^2+^ transfer by stimulating the calcium export receptor IP3R, boosting mitochondrial bioenergetics [2,29]. Our recent RNA-sequencing results indicate that OXPHOS is among the most significantly perturbed gene sets (Hallmark) in ERO1 knock-out MDA-MB231 breast tumors [58]. Although the effect of ERO1 on tumor bioenergetics still needs to be assessed in cancer settings *in vivo,* these findings suggest that ERO1 could take part in the metabolic rewiring of tumors by affecting OXPHOS, heightening the tumor aggressive phenotype.

## 5. ERO1 in tumor angiogenesis

Solid tumors adapt to hypoxic conditions by activating HIF-1, a transcription factor involved in *de novo* angiogenesis [33]. Angiogenesis in tumors refers to the formation of blood vessels, required to supply nutrients and oxygen to the growing biomass, leading to its metastatic spread [5,10]. A plethora of hypoxia-dependent growth factors and cytokines stimulate angiogenesis. The paradigmatic example of these factors is vascular endothelial growth factor A (VEGF-A) which triggers vessel formation [37,38]. Therefore, VEGF positively correlates with the vessels in tumor sections and thus, is a negative prognostic factor for survival [15,59,63].

Functionally, hypoxia raises the levels of angiogenic factors and impairs post-translational disulfide bond formation, affecting the function of disulfide bonded angiogenic factors [23]. In this way, hypoxia cooperates with ERO1 loss in impairing disulfide bond formation of angiogenic factors.

Our analysis of the secretome of highly aggressive TNBC MDA-MB231 cells cultured under hypoxic conditions identified a selective effect of the lack of ERO1 on the oxidative status of some cysteines and on the defective secretion of some disulfide-bonded proteins including HIF-1 targets involved in the vessel formation. This suggests that a subset of proteins is still oxidatively folded *via* ERO1 in hypoxic conditions [56], as will be highlighted next.

**Fig. 4.**
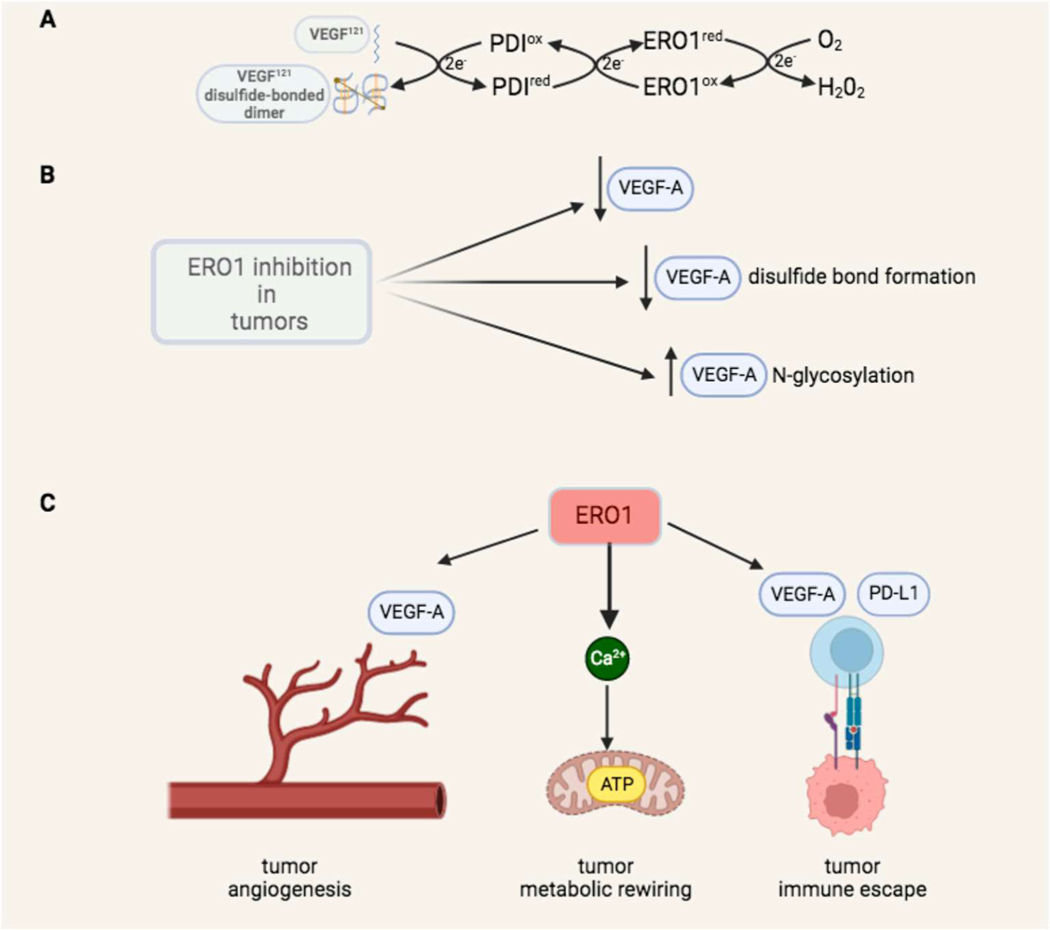
ERO1 effect on tumor angiogenesis, bioenergetics and immune escape. The couple ERO1 and PDI catalyzes the reaction of disulfide bond formation of new client proteins into ER. Our data suggest that ERO1 participates in the disulfide bond formation of VEGF^121^, an isoform of VEGFA, that contains three intramolecular disulfide bonds and is assembled into a disulfide-linked homodimer [20]. In the canonical reaction of disulfide bond formation, the electrons flow first from VEGF to the oxidized PDI and then to ERO1, that moves them to the final acceptor O_2_, which is reduced to H_2_O_2_. In this redox reaction, the final balance is the production of one molecule of H_2_O_2_ for each disulfide bond inserted in the client protein. ERO1 inhibition acts at multiple levels on VEGF: lowering its levels, impairing its disulfide bond formation and increasing its *N*-glycosylation [56–58]. ERO1 supports tumors through at least three mechanisms: by favoring tumor angiogenesis through regulation of VEGFA and other HIF-1 dependent angiogenic factors [58,56] inducing tumor metabolic rewiring thereby improving mitochondrial bioenergetics [2], and interfering with tumor immune surveillance, acting on VEGFA and PD-L1 [51].

VEGF^121^ is a secreted VEGF-A isoform and a HIF-l-dependent regulator of angiogenesis. VEGF^121^ binds its receptor, VEGF Receptor 2 (VEGFR2), as a disulfide-linked homodimer, activating the signal transduction which culminates in new vessel formation [35]. ERO1 loss slows the formation of functional disulfides in VEGF^121^. Under hypoxia, VEGF^121^ secretion from ERO1 knock-out cells was severely reduced, pointing to a failure in the compensation of ERO1 activity as protein disulphide oxidase in this condition of low oxygen (Fig. 4A), and to the dependency of VEGF^121^ folding and secretion from oxygen tinder conditions of ERO1 loss. It is still being investigated whether the effect of ERO1 on VEGF oxidative folding is mediated by PDI - following the canonical pathway of disulfide bond formation in proteins [74], or is direct (Fig. 4A).

We also pinpointed a feedback circuit between ERO1 and its upstream UPR mediators PERK and ATF4, which has been shown to increase VEGFA expression [62]. This hints at some indirect transcriptional control of ERO1 on VEGFA and might explain the lower levels of VEGFA mRNA in ERO1-deficient breast cancer cells [56].

A thought-provoking hypothesis suggests that ERO1-mediated H_2_O_2_ fluxes stabilize HIF-1 and the latter triggers VEGF [25]. However, this would imply either the uncontrolled passage of ERO1-generated H_2_O_2_ freely through the ER to stabilize HIF-1 in cytoplasm [70], or the involvement of a relay system [47] that transduces the H_2_O_2_ signal to HIF-1.

We also detected an effect of ERO1 loss on VEGF^121^ *N*-glycosylation. A unique *N*-glycosylation consensus lies within two of the intra-chain disulfide bridges of this VEGF isoform [20]. Lack of ERO1 boosts the interaction between VEGF^121^ and MAGT1, a thioredoxin-containing component of the STT3B oligosaccharyl-transferase complex, giving rise to *N*-hyperglycosylated VEGF^121^ in ERO1 KO cells [57].

The effect of ERO1 loss on protein *N*-glycosylation, in the case that was not only restricted to VEGF and that *N*-glycosylation was functionally relevant, might connect ERO1 to other cancer-promoting mechanisms different from angiogenesis, given the causal link between alterations in protein *N*-glycosylation and cancer [39,68].

These findings support the notion that in hypoxic conditions, inhibition of ERO1 restrains angiogenesis at various levels, by impairing VEGF, oxidative folding, and increasing its *N*-glycosylation (Fig. 4B).

The effect of ERO1 loss on VEGF impairment results in breast tumor and hepatocarcinoma cells with a lower pro-angiogenic potential [65], metastatic breast tumors with reduced blood vessels in the primary site and less lung metastases [56,70]. (Fig. 4C).

To conclude, ERO1 is emerging as one of the most interesting and versatile prototypes of angiogenic factors, on account of its multiple (transcriptional and post-translational) effects on the expression and correct assembly of VEGF and other HIF-l-dependent angiogenic factors.

## 6. ERO1 in tumor immune escape

Besides its pro-angiogenic function, VEGF has immune-suppressive properties, inhibiting the trafficking of tumor-reactive T cells to tumors [60]. As ERO1 regulates VEGF in TNBC, its inhibition may impair VEGF, enhancing the trafficking of tumor-reactive T cells to cancer and favoring immune surveillance [56,60].

Immune checkpoint regulators are cell surface proteins whose function is to control immune responses. PD-1 is expressed on activated B and T cells and, after binding to the two ligands PD-L1 and PD-L2, activates signal transduction, resulting in T cell exhaustion. PD-L1 is expressed in many tumors, from which it co-opts this pathway, leading to tumor-associated immune escape, with consequent tumor growth and spread [48].

Regarding the immune checkpoints, ERO1 up-regulates PD-L1 expression through protein oxidative folding and indirectly by up-regulating PD-L1 mRNA expression in human TNBC cell lines. Consequently, ERO1 knockdown can attenuate PD-L1-mediated T-cell apoptosis [51].

The immunosuppressive activity of PD-L1 is tightly modulated by *N*-glycosylation [28], and it will be interesting to study the effect of ERO1 loss on the *N*-glycosylation of PD-L1. ERO1 inhibition, on the one hand, might impair VEGF, enhancing the trafficking of tumor-reactive T cells to the tumor, while on the other, it might lower PD-L1 expression in tumors, reducing the related immunosuppressive mechanism (Fig. 4C). Collectively, these findings suggest that ERO1 influences cancer patients’ responses to immune therapy.

**Fig. 5.**
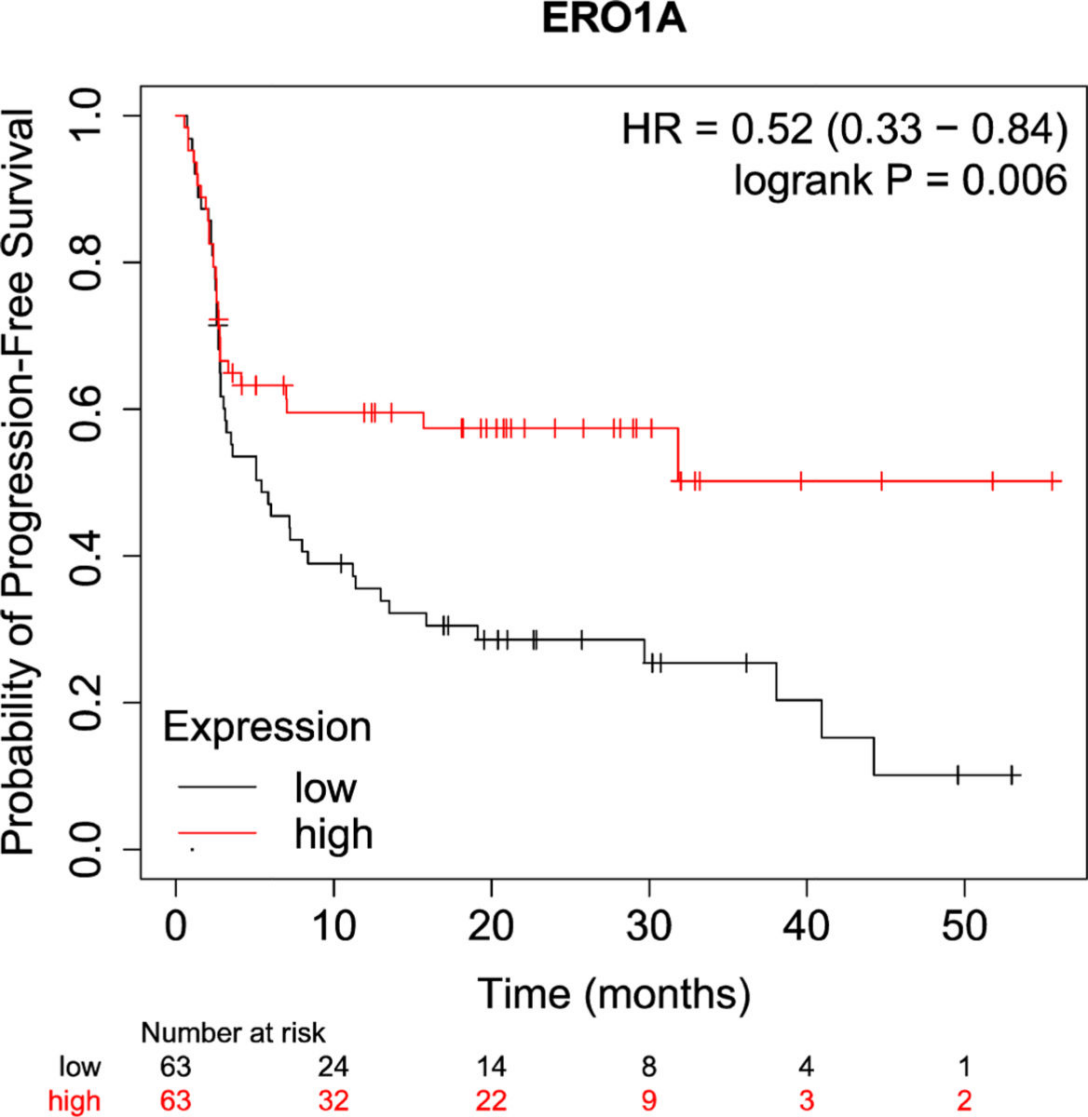
Correlation between ERO1 expression levels and responses of melanoma patients to immune check-point inhibitors (ICI). Kaplan-Meier plot (from Kaplan-Meier plotter) depicting progression-free survival (PFS) of melanoma patients receiving immunotherapy (*N* = 570) and stratified for ERO1 gene expression levels in quartiles (Q), then comparing the high ERO1 Ql, in red, *versus* low ERO1 Q4, in black. Statistical significance was assessed using a log-rank test. (For interpretation of the references to colour in this figure legend, the reader is referred to the web version of this article.)

Melanoma is the cancer most frequently treated with monoclonal antibodies against PD-1 (anti-PD-1), CTLA4 (anti-CTLA-4) and PD-L1 (anti-PD-Ll). These antibodies work as immune checkpoint inhibitors (ICI), enhancing the anti-tumoral properties of T cells and offering clinical efficacy in metastatic melanoma. In fact, they provide long-term durable cancer control in nearly 50% of patients, compared with less than 10% previously [26]. Given the frequent use of ICI for the treatment of melanoma and positive clinical outcomes, online data correlating gene levels and ICI response in melanoma are already publicly available. We therefore analyzed ERO1 levels and response to ICI in melanoma, studying progression-free survival (PFS) of melanoma patients treated with ICI and stratified for gene expression levels of ERO1, with the KM Plotter tool (which is publicly available at https://kmplot.com/analysis). Melanoma patients with high ERO1 levels had good rates of PFS, suggesting that ERO1 levels affect their response to immunotherapy (Fig. 5). Therefore, high ERO1 levels, by increasing immune checkpoints, favor tumor immune escape on one hand while favoring the clinical efficacy of ICI on the other.

It was recently reported that ERO1 induces tumor immunosuppression in mouse models of B16 melanoma, Lewis lung cancer, and MC-38 colon cancer and enhances the efficacy of PD-1 antibody immunotherapy. However, at variance with the data from KM Plotter tool, the same paper suggested high PFS in a cohort of melanoma patients with low ERO1 levels [30].

In conclusion, these findings suggest that selective targeting of ERO1’s action toward a subset of its downstream effectors has the potential to dampen tumor angiogenesis, metabolic rewiring and immune tolerance (Fig. 4C).

## 7. ERO1 inhibition in cancer

Regarding the importance of ERO1 inhibition in counteracting tumor growth and dissemination, ERO1 genetic inhibition impairs TNBC resilience and its resistance to angiogenic and chemo-therapy, through a synergic effect in blunting VEGF secretion and impairing proteostasis [56,58]. Our studies in preclinical animal models of TNBC suggest that ERO1 genetic inhibition improves the efficacy of a VEGF monoclonal antibody (B20) in restraining the tumor and metastasis as well as the cytotoxic effect of the protein translational activator ISRIB [45,56,58]. In TNBC, chronic ERO1 inhibition, on the one hand, impairs VEGF and other angiogenic factors, thus rendering tumors more responsive to VEGF antibody-based anti-angiogenic therapy. On the other hand, chronic ERO1 inhibition activates PERK branch of UPR, thereby adapting tumors to live with a low client protein load in hypoxia. Although ISRIB does not have any direct or indirect effect on ERO1 (which is not regulated by CHOP in this context), the rapid ISRIB-dependent increase in protein translation triggers proteotoxicity and results in cytotoxic responses in cells deficient for ERO1, an enzyme with protein folding activity [56].

The UPR was suggested as a mechanism of resistance to paclitaxel, one of the first-line drugs for breast cancer [27]. RNA sequencing data suggest opposite responses of WT and ERO1 KO breast cancers to the combination ISRIB and paclitaxel on the UPR pathway: the UPR is upregulated in WT tumors treated with the combination but is down-regulated in ERO1 KO tumors treated with this combination of drugs, suggesting that the resistance to paclitaxel is blunted by ERO1 deficiency [58].

Despite evidence of the importance of inhibiting ERO1 in tumors, very few studies have pursued identifying and developing ERO1 inhibitors. EN460 is a known ERO1 inhibitor which came, together with the structurally similar QM295, from high-throughput screening of a library containing 210,965 compounds. It inhibits ERO1 *in vitro* and *in vivo* in a low micromolar range [3]. Numerous lines of evidence suggest that at least one cysteine residue generated during activation and/or catalytic turnover of ERO1 is a target of EN460. *in vivo,* EN460 leads to the trapping of ERO1 in a reduced state, inactivating the enzyme [3]. Further molecular docking studies suggest that EN460 binds to the FAD pocket of ERO1 through hydrogen bonding and hydrophobic interactions [17]. Adduct formation between ERO1 and EN460 leads to significant weakening of the binding to the FAD prosthetic group, suggesting that exposure to EN460 leads to loss of the FAD from the holoenzyme, hence to the loss of enzymatic activity.

EN460 contains an enone function, which is a potent electrophile Michael acceptor that could interact with abundant thiol-containing compounds such as DTT and GSH. On the basis of this potential lack of selectivity, compounds belonging to the class of covalent inhibitors containing an electrophile, such as a Michael acceptor, have been neglected in clinical use. Until recently, these compounds were shunned by the pharmaceutical industry on account of concerns about off-target effects and potential toxicity. However, current interest has been aroused by the clinical success of targeted covalent inhibitors (TCI) in cancer therapy, with eight drugs approved in the past decade [46]. Based on the fact that EN460 was selected as an ERO1 inhibitor from a library containing other Michael acceptors and that EN460 inhibits ERO1 *in vivo,* we argue against an overall lack of specificity of EN460 toward ERO1. We suggest that lead compound optimization of EN460 aimed at improving some characteristics such as the selectivity toward ERO1 - increasing its potency, and the lack of solubility in aqueous solution - which is a significant impediment for *in vivo* studies - might speed up the preclinical validation of ERO1 inhibitors as new anti-cancer drugs.

On claiming off-target effects of EN460, a new ERO1 inhibitor, B12, recently emerged from *in vitro* screening of 5800 compounds based on ERO1 activity. Further derivatives of B12 were screened and B12–5 was identified as an ERO1 inhibitor with an IC50 *in vitro* very similar to EN460 [21]. However, due to the lack of any Michael acceptor in B12 and its derivative B12–5, the mechanism of ERO1 inhibition is puzzling. Furthermore, the lack of evidence of ERO1 inhibition *in vivo,* the lack of any improved selectivity, and the still limited water solubility question any real advantage for its use in (pre)clinical settings.

## 8. Conclusion

In the present essay, we highlighted recent studies demonstrating the upregulation of ERO1 across multiple human tumors in association with poor survival. Pre-clinical studies in mice pointed to a contributory role of ERO1 in tumor growth and metastasis. The observations that mammals can compensate for the loss of ERO1 activity, suggest that non-tumor cells might safely survive under conditions wherein ERO1 is inhibited. Despite being dispensable in non-tumor cells, ERO1 is essential for the fitness of tumor cells under hypoxic conditions, interfering with tumor angiogenesis, metabolic rewiring, and immune escape. Thus, these recent findings collectively offer the prospect of targeting this enzyme with inhibitors in tumor cells where ERO1 activity is essential for growth. ERO1 is now emerging as one of the most interesting and versatile prototypes of anti-cancer and angiogenic factors, and an ERO1 inhibitor might pave the way for new lines of intervention in cancer treatment.

## Data availability

No data was used for the research described in the article.

## Figures and Tables

**Table 1:** indicating ERO1 mutations in different cancer types (TCGA Pan Cancer ATLAS).

Study of Origin	Sample ID	Protein Change	Mutation Type

Bladder Urothelial Carcinoma (TCGA, PanCancer Atlas)	TCGA-XF-A8HG-01	D160N	Missense_Mutation
Bladder Urothelial Carcinoma	TCGA-BT-A42F-01	P220R	Missense_Mutation
Bladder Urothelial Carcinoma	TCGA-K4-A6FZ-01	Q285*	Nonsense_Mutation
Bladder Urothelial Carcinoma	TCGA-FD-A6TE-01	P355S	Missense_Mutation
Bladder Urothelial Carcinoma	TCGA-ZF-A9R0-01	E458Q	Missense_Mutation
Brain Lower Grade Glioma	TCGA-DU-8165-01	T148A ERO1A-GPR137C	Missense_Mutation
Breast Invasive Carcinoma	TCGA-C8-A26V-01	Fusion ERO1A-TXNDC16	fusion
Breast Invasive Carcinoma	TCGA-C8-A26V-01	Fusion	fusion
Breast Invasive Carcinoma	TCGA-A8-A08L-01	I53V	Missense_Mutation
Breast Invasive Carcinoma	TCGA-B6-A0WV-01	V109I	Missense_Mutation
Breast Invasive Carcinoma	TCGA-BH-A0C0-01	N361D	Missense_Mutation
Cervical Squamous Cell Carcinoma	TCGA-*VS*-A8EJ-01	Q225*	Nonsense_Mutation
Colorectal Adenocarcinoma	TCGA-AA-3510-01	K67T	Missense_Mutation
Colorectal Adenocarcinoma	TCGA-AZ-4315-01	X78_splice	Splice_Region
Colorectal Adenocarcinoma	TCGA-AG-A002-01	L240I	Missense_Mutation
Colorectal Adenocarcinoma	TCGA-AA-3977-01	R245I	Missense_Mutation
Colorectal Adenocarcinoma	TCGA-QG-A5Z2-01	L254P	Missense_Mutation
Colorectal Adenocarcinoma	TCGA-AZ-4315-01	I388T	Missense_Mutation
Head and Neck Squamous Cell Carcinoma	TCGA-CV-A6K2-01	E167K	Missense_Mutation
Head and Neck Squamous Cell Carcinoma	TCGA-CN-6021-01	R449T	Missense_Mutation
Kidney Renal Clear Cell Carcinoma	TCGA-A3-3308-01	K375T	Missense_Mutation
Liver Hepatocellular Carcinoma	TCGA-4R-AA8I-01	E243G	Missense_Mutation
Lung Adenocarcinoma	TCGA-55-8302-01	R64I	Missense_Mutation
Lung Adenocarcinoma	TCGA-95-7947-01	W155C	Missense_Mutation
Lung Adenocarcinoma	TCGA-50-5072-01	W197R	Missense_Mutation
Lung Adenocarcinoma	TCGA-50-6590-01	E346*	Nonsense_Mutation
Lung Adenocarcinoma	TCGA-97-8172-01	A365Cfs*4	Frame_Shift_Ins
Lung Squamous Cell Carcinoma	TCGA-56-8305-01	S71N	Missense_Mutation
Lung Squamous Cell Carcinoma	TCGA-66-2791-01	E274Q	Missense.Mutation
Lung Squamous Cell Carcinoma	TCGA-6 A-AB49-01	A446V	Missense_Mutation
Ovarian Serous Cystadenocarcinoma	TCGA-61-2012-01	E129K	Missense_Mutation
Ovarian Serous Cystadenocarcinoma	TCGA-61-1919-01	R287*	Nonsense_Mutation
Ovarian Serous Cystadenocarcinoma	TCGA-29-1781-01	V318A	Missense_Mutation
Pancreatic Adenocarcinoma	TCGA-IB-7652-01	L457*	Nonsense_Mutation
Skin Cutaneous Melanoma	TCGA-D9-A149-06	V39A	Missense_Mutation
Skin Cutaneous Melanoma (	TCGA-FW-A3R5-06	P84L	Missense_Mutation
Skin Cutaneous Melanoma	TCGA-WE-A8ZT-06	P103S	Missense_Mutation
Skin Cutaneous Melanoma	TCGA-FS-A1ZR-06	El 46*	Nonsense_Mutation
Skin Cutaneous Melanoma	TCGA-EE-A17X-06	L153F	Missense.Mutation
Skin Cutaneous Melanoma	TCGA-GN-A262-06	P174H	Missense_Mutation
Skin Cutaneous Melanoma	TCGA-EE-A3AD-06	G190C	Missense_Mutation
Skin Cutaneous Melanoma	TCGA-EE-A29M-06	P355S	Missense_Mutation
Skin Cutaneous Melanoma	TCGA-FR-A726-01	P355S	Missense_Mutation
Skin Cutaneous Melanoma	TCGA-EE-A2GU-06	S362L	Missense_Mutation
Thyroid Carcinoma	TCGA-BJ-A0ZH-01	Y76C	Missense_Mutation
Uterine Corpus Endometrial Carcinoma	TCGA-B5-A0JY-01	R55I	Missense_Mutation
Uterine Corpus Endometrial Carcinoma	TCGA-AJ-A3EL-01	K78T	Missense_Mutation
Uterine Corpus Endometrial Carcinoma	TCGA-F1-A2F4-01	X145_splice	Splice_Region
Uterine Corpus Endometrial Carcinoma	TCGA-B5-A1MX-01	F165L	Missense_Mutation
Uterine Corpus Endometrial Carcinoma	TCGA-EO-A22X-01	E167*	Nonsense_Mutation
Uterine Corpus Endometrial Carcinoma	TCGA-B5-A0JY-01	R187H	Missense_Mutation
Uterine Corpus Endometrial Carcinoma	TCGA-BK-A0CC-01	D195Y	Missense_Mutation
Uterine Corpus Endometrial Carcinoma	TCGA-B5-A3FA-01	E205K	Missense_Mutation
Uterine Corpus Endometrial Carcinoma	TCGA-BS-A0UV-01	E205K	Missense_Mutation
Uterine Corpus Endometrial Carcinoma	TCGA-AX-A05Z-01	E206*	Nonsense.Mutation
Uterine Corpus Endometrial Carcinoma	TCGA-DI-A1BU-01	C208R	Missense_Mutation
Uterine Corpus Endometrial Carcinoma	TCGA-EO-A22R-01	S228R	Missense.Mutation
Uterine Corpus Endometrial Carcinoma	TCGA-AX-A05Z-01	E230*	Nonsense_Mutation
Uterine Corpus Endometrial Carcinoma	TCGA-A5-A0G2-01	R245I	Missense_Mutation
Uterine Corpus Endometrial Carcinoma	TCGA-EO-A22X-01	K275N	Missense_Mutation
Uterine Corpus Endometrial Carcinoma	TCGA-E6-A1M0-01	I291M	Missense_Mutation
Uterine Corpus Endometrial Carcinoma	TCGA-B5-A11E-01	Q336*	Nonsense_Mutation
Uterine Corpus Endometrial Carcinoma	TCGA-EO-A22U-01	K368N	Missense_Mutation
Uterine Corpus Endometrial Carcinoma	TCGA-BK-A6W3-01	R379*	Nonsense_Mutation
Uterine Corpus Endometrial Carcinoma	TCGA-DF-A2KU-01	R379Q	Missense_Mutation
Uterine Corpus Endometrial Carcinoma	TCGA-EO-A22X-01	R383I	Missense_Mutation
Uterine Corpus Endometrial Carcinoma	TCGA-F1-A2D0-01	K396T	Missense_Mutation
Uterine Corpus Endometrial Carcinoma	TCGA-B5-A1MR-01	K413N	Missense_Mutation
Uterine Corpus Endometrial Carcinoma	TCGA-AX-A2HD-01	A446V	Missense_Mutation
Uterine Corpus Endometrial Carcinoma	TCGA-F1-A2F4-01	S453N	Missense_Mutation
